# Case report: A rare case of three synchronous colon tumors with metastasis to the left inguinal lymph node

**DOI:** 10.3389/fonc.2024.1459620

**Published:** 2025-01-07

**Authors:** Roxana Loriana Negrut, Adrian Cote, Ovidiu Tica, Adrian Marius Maghiar

**Affiliations:** ^1^ Department of Medicine, Doctoral School of Biomedical Sciences, Faculty of Medicine and Pharmacy, University of Oradea, Oradea, Romania; ^2^ Department of General Surgery I, County Clinical Emergency Hospital Bihor, Oradea, Romania; ^3^ Department of Surgical Disciplines, Faculty of Medicine and Pharmacy, University of Oradea, Oradea, Romania; ^4^ Department of Morphologic Disciplines, Faculty of Medicine and Pharmacy, University of Oradea, Oradea, Romania

**Keywords:** colon cancer, rare metastasis, synchronous tumors, inguinal metastasis, cancer pathway, metastasis (cancer metastasis), multiple tumors

## Abstract

This study presents a rare case of three synchronous colon tumors with metastasis to the left inguinal lymph node, challenging the conventional understanding of the metastatic pathways and highlighting the exceptional nature of such occurrences. This highlights the importance of considering alternative atypical metastatic routes for the management of colon cancer. A literature search was performed to identify similar cases. Our findings emphasize the need for broader diagnostic evaluations to detect atypical metastasis at early stages. Furthermore, this case highlights the need for potential refinements in follow-up and screening protocols to capture unusual patterns of cancer spread. This case encourages further research into innovative treatments tailored to the unique metastatic behaviors observed in synchronous colon tumors, with a view to improving outcomes in similar cases.

## 1 Introduction

Synchronous colon tumors are characterized by the Warren and Gates criteria (1) as the presence of multiple histologically distinct tumors in the colon that occur in one patient at the same time or within 6 months (2). This pathology is considered uncommon, although recent studies have shown an increasing prevalence, reaching up to 17% (2, 3). Surgical resection is the primary treatment for synchronous colon tumors (4). Existing literature confirms that synchronous colorectal cancer has a male predominance without any obvious reason for this entity (5, 6). A large-scale retrospective study published in 2017, conducted between July 2003 and December 2010 on patients with synchronous colorectal cancer who received surgical treatment, highlighted that synchronous colon cancer is different from single-site cancer in terms of patient demographics, tumor location, and pathologic features, requiring different surgical treatment plans, which can be divided into more regional resections or extensive resections (7). By comparing the two surgical paths, they identified that two regional resections were more suitable (7).

However, synchronous colon tumors involve two distinct histopathological subtypes combined with contralateral inguinal lymph node metastasis. In this article, we present a case of three synchronous tumors located in the right colon with contralateral inguinal lymph node metastasis, challenging the conventional understanding of metastatic pathways and underscoring the need for reconsideration of management strategies in such cases.

## 2 Case description

A 66-year-old man with type II diabetes and cardiac comorbidities presented to the emergency department (ED) with pain in the left inguinal region. Local clinical examination showed a lump in the left inguinal region that was painful both at rest and to palpation and could not be pushed back into place using the standard manual method known as the “taxis maneuver.” The presumptive diagnosis was strangulated inguinal hernia. Emergency ultrasonography (US) was performed, but the results were inconclusive. Blood tests showed that the patient had anemia (low hemoglobin level, 8.53 g/dl) and slightly low levels of proteins (hypoproteinemia, with total protein of 5.2 g/dl). Surgery was decided upon as the next step.

During surgery, a mass measuring approximately 8 cm × 6 cm was found after performing an incision in the left inguinal region. The mass was firm to the touch and extended deep into the surrounding tissue. The mass was surgically removed, and subsequent histopathological examination revealed tumoral proliferation with extensive necrotic areas, which was interpreted as metastasis of low-grade adenocarcinoma originating from the digestive tract. The patient did not return for follow-up care and the pathology results were not retrieved.

Three months later, the patient returned to the ED with symptoms of lower abdominal pain, weight loss (15 kg in 4 months), and nausea. He was admitted to the Gastroenterology Department, where esophagogastroduodenoscopy and colonoscopy were performed along with another US examination. Ultrasonography showed slow movement and distension of the small intestine. Upper Gastrointestinal Endoscopy (UGE) revealed inflammation in the duodenum (D2 section), and biopsy confirmed chronic inflammation. A colonoscopy revealed a colon obstruction in the transverse colon that could not be passed through the endoscope. The patient was treated by the General Surgery Department for emergency surgery due to bowel obstruction. A plain abdominal radiograph obtained before surgery confirmed the presence of multiple levels of fluid and gas, indicating a blockage, but without evidence of free air in the abdominal cavity. Due to acute renal failure, CT could not be performed.

The patient underwent emergency surgery during which a midline incision was made to open the abdomen. Upon inspection of the abdominal cavity, it was found that the small intestine was distended; at the site of the cecum, a 6/5 cm tumor was found, with an irregular shape and firm texture. Moreover, moving forward with the colon examination, another 9 cm tumor with the same macroscopic aspect and texture was discovered. No visible signs of direct invasion of the abdominal wall of cancer spreading to other organs were observed. Extended right hemicolectomy was performed with ileocolic anastomosis end to side using manual stitching in a single layer.

**Figure 1:**
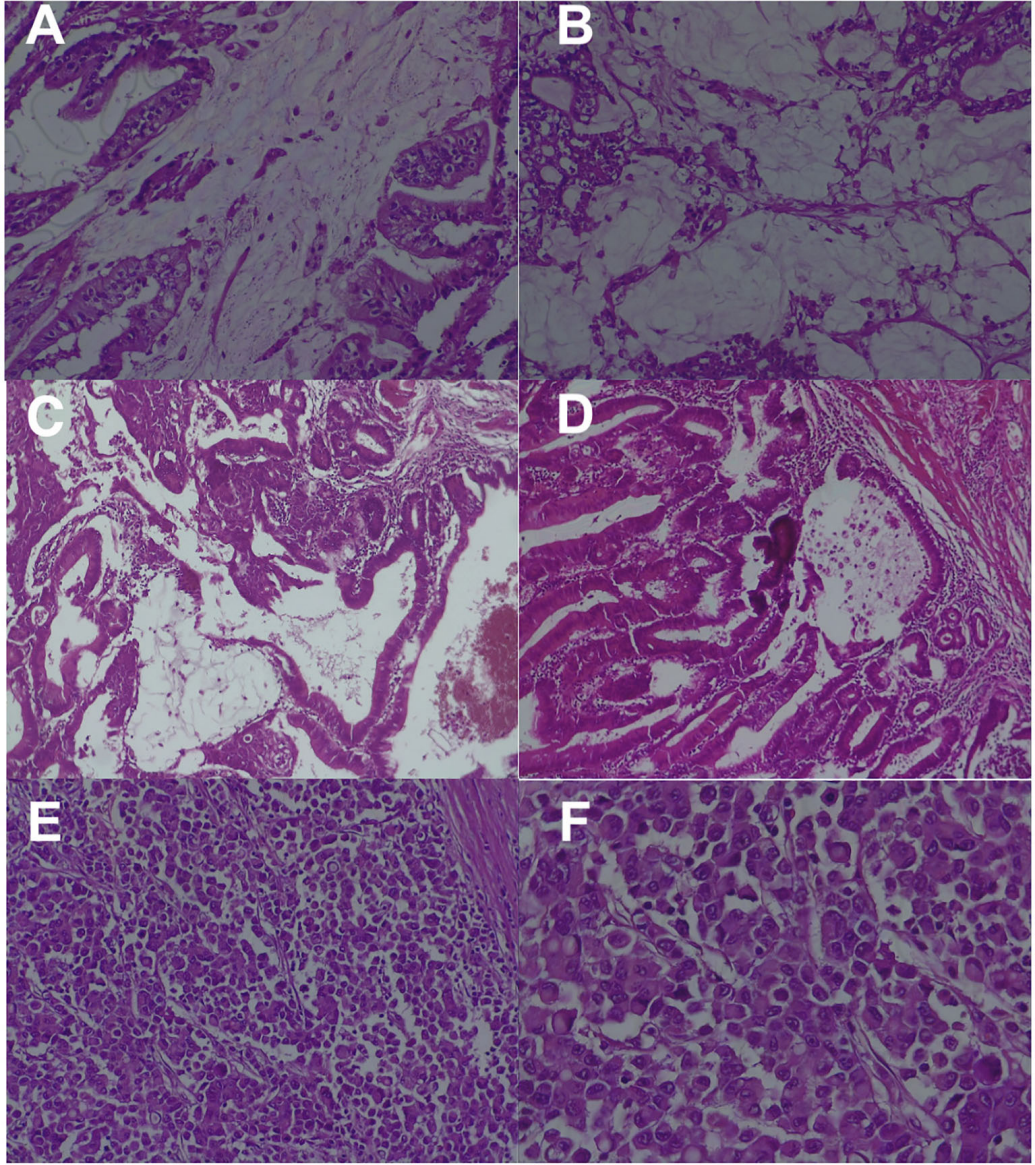
Histopathological findings **(A)** Hematoxylin–eosin stain, ×20: Tumor A with features of cecal mucinous adenocarcinoma, moderately to poorly differentiated; **(B)** Hematoxylin–eosin stain, ×20: Cecal tumor A with pools of mucin in which tumor cells are floating with fibrous septa; **(C)** Hematoxylin–eosin stain, ×10: Cecal tumor B with features of intramucosal adenocarcinoma; **(D)** Hematoxylin–eosin stain, ×20: Cecal tumor B showing tumor glands in the inflammatory stroma with signs of *in situ* malignancy; **(E)** Hematoxylin–eosin stain, ×20: Transverse colon tumor with features of signet ring cell carcinoma invading the pericolonic tissue; **(F)** Hematoxylin–eosin stain, ×40: Transverse colon tumor showing discohesive ‘signet ring’ cells in the extramural component.

The histopathological report shows three synchronous tumors, all removed with free resection margins (both cut ends and the non-peritonealized resection margin):

The first cecal tumor measured 60 mm in its largest diameter and had a mucinous component of over 55%; it involved the tissues surrounding the colon (pT3) and had 30% tumor necrosis. It invaded nearby blood vessels and lymph nodes, with two positive nodes out of the 14 harvested. (pT3N1bG3L1R0). Shown in **Figures 1A, B**.

The second cecal tumor was 40 mm in size and showed histological features suggestive of intraepithelial carcinoma (pTis). See **Figures 1C, D**.

The third tumor was located in the transverse colon with a histological pattern of signet ring cell carcinoma (pT3) with extensive intratumoral necrosis and 19 out of 24 positive nodes harvested. Extranodal tumor growth and lymphatic and perineural invasion were also observed (pT3N2bG3L1Pn1). See **Figures 1E, F**.

The patient recovered favorably and was discharged one week later. However, he did not present for follow-up appointments for the next 8 months.

**Figure 2:**
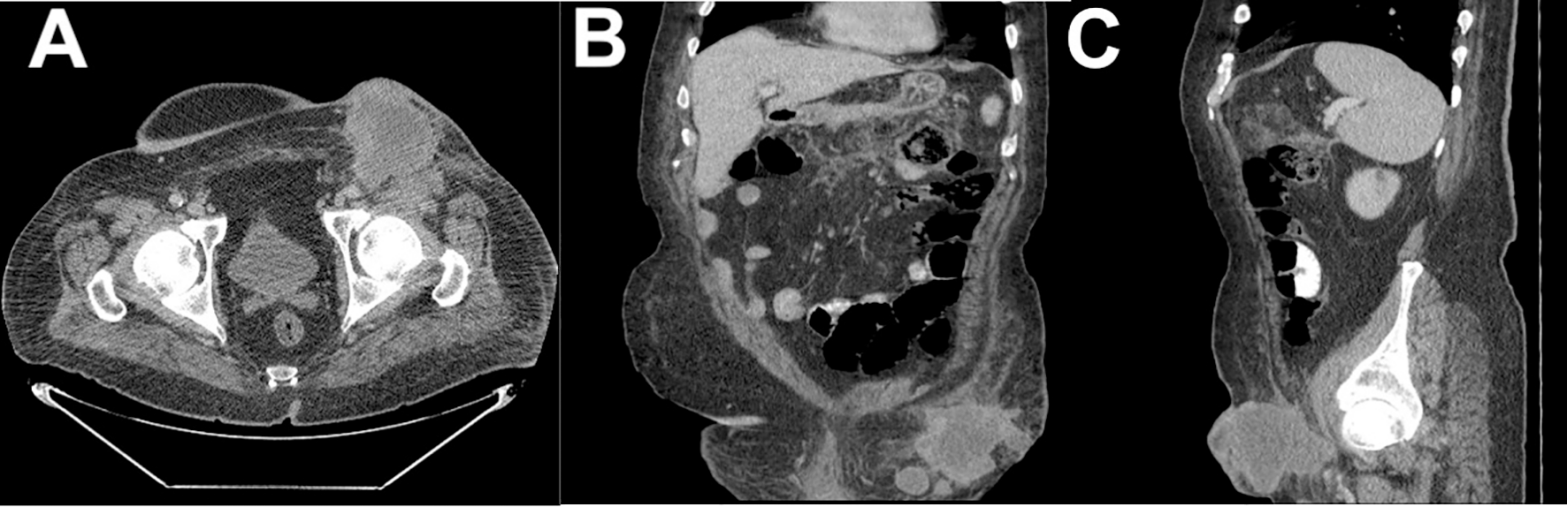
CT scan of the left inguinal mass: **(A)** axial plane view; **(B)** coronal plane view; and **(C)** sagittal plane view.

Eight months after surgery, the patient returned to the ED because of a recurrence of the inguinal tumor, which had necrotic tissue and pus. A CT scan revealed a necrotic expansive tumoral mass with air-filled cavities and interruption in the continuity of the skin in the left groin area, with a diameter of 4.6/15 cm, encompassing the common femoral vascular bundle and the emergence of the femoral artery, and extending toward the pectineus muscle and the distal segment of the iliac muscle with an infiltrative aspect. The scan also showed necrotic lymph node blocks around the aorta, along the common and iliac vascular bundles bilaterally, with diameters of up to 25/35 mm. The CT scans are shown in **Figure 2** (A) axial plane, (B) coronal plane and (C) sagital plane.

Given the extensive spread of the disease, the patient’s condition was considered inoperable, and the patient was referred to the oncology department for further management.

## 3 Discussion

According to Globocan, in 2022 colorectal cancer in Romania ranked first in incidence (13.541 cases, 12.9%) and second in mortality (7,381 cases, 13.1%) (8). The prevalence of synchronous tumors ranged from 1.1% to 8.1% (6). However, after a brief literature search, we found that extremely rare cases of colon tumors can result in inguinal node metastasis. The usual sites for colorectal cancer metastasis include the liver and lungs, but a few cases have spread to rare sites such as the spleen, muscle, heart, and inguinal lymph nodes (9–14).

In this patient, the development of three synchronous colon tumors accompanied by metastasis in an inguinal lymph node was an atypical presentation. The ways by which colon cancer can spread to the inguinal lymph nodes are uncertain and not well understood, which makes this case particularly noteworthy. Unlike typical cases in which the lymphatic spread of colon cancer follows the superior and inferior mesenteric arteries, this case suggests an alternative route, possibly through the abdominal wall. However, the precise mechanism remains uncertain and warrants further investigation.

## 4 Literature review

A literature search of the Web of Science database and references cited in the relevant articles revealed that only 10 other cases of inguinal lymph node metastasis from colon carcinoma have been reported. These cases, summarized in **Table 1**, provide insight into the variability of tumor location, histology, and possible metastatic pathways.

**Table 1:** Cases published in the literature of colon carcinoma and inguinal lymph node metastasis.

Author	Year of publication	Gender	Colon tumor site	Side of the inguinal lymph node	pTNM stage	Histologic type
Tanabe et al. (15)	2019	Female	Sigmoid colon	Both	pT3N2bM1	Well-differentiated adenocarcinoma
Neirouz et al. (16)	2024	Male	Ascending colon	Right	pT3N1aM0	Well-differentiated adenocarcinoma
McGraw (17)	2011	Male	Rectosigmoid colon	Right	Not mentioned	Moderately differentiated invasive colon carcinoma
Alzahrani et al. (14)	2020	Male	Splenic flexure	Right	Not mentioned	Moderately differentiated mucinous adenocarcinoma
Kintaro et al. (18)	2017	Female	Ascending colon	Right	T3N0M1	Well-differentiated adenocarcinoma
Pisanu et al. (19)	2011	Male	Sigmoid colon	Left	pT4N1M0	Adenocarcinoma
Hakeem, et al. (20)	2009	Female	Cecum	Left	pT3N2M1	Moderately differentiated adenocarcinoma
Hara et al. (21)	2012	Male	Cecum	Right	T4N1M0	Mucinous carcinoma
Iwamoto et al. (22)	2014	Female	Sigmoid colon (colostomy after benign rectal tumor)	Left	T4N0M1	Moderately differentiated adenocarcinoma
Wu et al. (23)	2009	Male	Hepatic flexure	Bilateral	Not mentioned	Adenocarcinoma

The case presented by Tanabe et al. focused on a rare case of inguinal lymph node metastasis from sigmoid cancer that developed 3 years after surgery and chemotherapy. They mentioned that inguinal lymph node metastasis is uncommon due to the typical lymphatic spread patterns of colon cancer, which follow the superior or inferior mesenteric arteries. They suggested that the pathway in this case originated from an abdominal wall metastasis (15).

Neirouz et al. reported a rare case of isolated inguinal lymph node metastasis from adenocarcinoma of the ascending colon in a 76-year-old patient found 2 years post treatment (surgery and adjuvant chemotherapy), mentioning that this kind of metastasis is extremely uncommon due to the route of the lymphatic drainage of the colon (16).

McGraw et al. reported a case of colon adenocarcinoma with bilateral inguinal metastases. The study theorizes that the spread may have been facilitated by postsurgical vascular formation or by the involvement of the anterior abdominal wall by micrometastasis (17).

Alzahrani and Alshehri presented a case of splenic flexure carcinoma with multiple splenic focal lesions, multiple peritoneal deposits, and right-sided inguinal metastasis, which is the first case to originate from the splenic flexure (14).

Another case report presented in the literature by Kintaro et al. was a patient with an ascending colon tumor with right inguinal lymphatic metastasis without evidence of regional lymph node metastasis or extramural tumor deposits, concluding that more similar cases are needed to properly clarify an effective treatment for such cases (18).

The study published by Pisanu et al. presented a rare case of isolated inguinal lymph node metastasis found 33 months after surgery for sigmoid colon adenocarcinoma invading the abdominal wall and adjacent colon lymph nodes. The authors suggested that the spread occurs through a lymphatic pathway along the left inferior epigastric artery due to the initial invasion of the abdominal wall (19).

Hakeem et al. discussed a rare case of cecal tumor with infiltration into the adjacent peritoneal fat tissue and a large left inguinal lymph node metastasis, mentioning that metastasis to unilateral or contralateral groin nodes is a rare phenomenon, emphasizing the need to consider atypical presentations of neoplasia in the differential diagnosis of inguinal lymphadenopathy. To the best of our knowledge, cecal cancer had not been reported to metastasize to inguinal nodes prior to this case reported in 2009 (20).

The case report presented by Hara et al. showed that inguinal lymph node metastasis developed three years after surgery for cecal cancer with abdominal wall invasion, suggesting that lymphatic flow from the abdominal wall is a possible route for metastasis to reach the inguinal nodes (21).

Iwamoto et al. present a unique case of inguinal node metastasis from an adenocarcinoma arising at a colostomy site, located in the sigmoid colon in a patient who had undergone abdominoperineal rectal resection 27 years earlier for a benign rectal tumor (22).

Wu et al. discussed a case of carcinoma of the right colon with a Sister Mary Joseph nodule and inguinal nodal metastasis, discussing the potential mechanisms for this uncommon form of cutaneous metastasis and addressing the unusual occurrence of colon cancer metastasizing to the inguinal lymph nodes, providing anatomical features and lymphatic drainage patterns (23).

The present case, in conjunction with the 10 cases from the literature, underscores the importance of recognizing atypical metastatic routes, such as inguinal lymph node involvement in colon cancer, suggesting that broader diagnostic evaluations should be considered in similar cases, potentially leading to the earlier detection of this atypical metastasis. Further research into tumor biology and the mechanisms of metastasis is needed to understand how cancer cells spread to atypical sites. Understanding these mechanisms may reveal new targets for therapeutic interventions and prevention strategies to improve patient prognosis.

## 5 Conclusions

This case report and the cases presented in the literature confirm that there may be unidentified or less-understood pathways of cancer spread. This could imply that, under certain conditions, cancer cells may find alternative routes for dissemination, such as involving the hypogastric pathway and nodes along the external iliac, common iliac, and para-aortic groups, leading to unusual metastatic sites such as inguinal lymph nodes. To our knowledge, this is the first case of three synchronous colon (cecum and transverse colon) tumors with contralateral inguinal lymph node metastasis presented in the existing literature.

Future research should focus on investigating the cellular mechanisms that allow colorectal cancer cells to spread through other atypical routes, such as the hypogastric pathway, and establish treatment protocols for these rare cases. Comparative large-scale studies of typical and atypical metastasis in colon cancer could clarify the factors that influence the spread to rare sites, such as inguinal lymph nodes.

We believe that this case could inspire research into innovative treatment modalities, leading to improved management strategies for metastatic cancers with atypical spread.

## Data Availability

The original contributions presented in the study are included in the article/supplementary material. Further inquiries can be directed to the corresponding author.
